# States as Sites of Educational (In)Equality: State Contexts and the Socioeconomic Achievement Gradient

**DOI:** 10.1177/2332858419872459

**Published:** 2019-09-03

**Authors:** Heewon Jang, Sean F. Reardon

**Affiliations:** Stanford University

**Keywords:** socioeconomic achievement gap, between-district income segregation, Stanford Education Data Archive (SEDA)

## Abstract

Socioeconomic achievement gaps have long been a central focus of educational research. However, not much is known about how (and why) between-district gaps vary among states, even though states are a primary organizational level in the decentralized education system in the United States. Using data from the Stanford Education Data Archive (SEDA), this study describes state-level socioeconomic achievement gradients and the growth of these gradients from Grades 3 to 8. We also examine state-level correlates of the gradients and their growth, including school system funding equity, preschool enrollment patterns, the distribution of teachers, income inequality, and segregation. We find that socioeconomic gradients and their growth rates vary considerably among states, and that between-district income segregation is positively associated with the socioeconomic achievement gradient.

## Introduction

The association between family socioeconomic status (SES) and children’s academic performance—the socioeconomic achievement gap—is large and has grown in the United States in the past few decades (Hanushek, Peterson, Talpey, & Woessmann, 2019; Reardon, 2011b).^1^ This is likely due, at least in part, both to widening income inequality and to an increased focus on educational success as central to economic success. In combination, these trends have led to increases in economic segregation (Owens, 2016; Reardon, Bischoff, Owens, & Townsend, 2018) and growing disparities in parental investments in children’s education (Duncan & Murnane, 2011; Kornrich & Furstenberg, 2013). As a result, rich and poor children today grow up in families and neighborhoods that provide very different levels of educational opportunities and supports than was the case 50 years ago. These socioeconomic disparities and the corresponding socioeconomic achievement gap are key features of today’s landscape of educational opportunity.

A great deal of literature on socioeconomic disparities in educational outcomes has focused on family, neighborhood, and school factors shaping achievement gaps (Reardon, 2011b; Sirin, 2005). This is true of much of the literature in the sociology of education since the Coleman report (Coleman et al., 1966). The role of school district context, and of between-district inequality and the state-level forces that shape such inequality, has been less central in the sociological literature, however. This is less true in the economics and public policy literatures, where there is considerable scholarship on the role of state and local school funding distribution policies and early childhood education policies. Some of this literature suggests that state-level policies are important in shaping local educational contexts and educational inequality (Jackson, Johnson, & Persico, 2016; Lafortune, Rothstein, & Schanzenbach, 2018; Valentino, 2018; Wong, Cook, Barnett, & Jung, 2008). This is unsurprising. States are key organizational levels in the decentralized education system in the United States. They set policy, distribute the large majority of public school funds, license teachers, and oversee curricula and accountability. Districts in turn have considerable leeway in allocating funds, assigning teachers and students to schools, and setting local priorities and foci. Both states and districts play roles in determining the availability and quality of pre-school programs, which affect students’ school readiness. Furthermore, between-district income segregation has grown in recent decades (Owens, 2016; Owens, Reardon, & Jencks, 2016), implying that between-district resource differences may become increasingly salient in shaping socioeconomic disparities in educational opportunities.

Consequently, it is useful to investigate whether and how state-level processes and contexts shape between-district academic outcome disparities. State processes play a role in moderating the effects of family and community differences in socioeconomic resources, given that states operate as a primary political division for the distribution of socioeconomic and educational resources. As such, they may function to equalize or exacerbate the achievement disparities that result from differences in family and community socioeconomic resources.

In this article, we examine how state-level policies and contexts are associated with between-district socioeconomic achievement gradients. We first define a state’s socioeconomic achievement gradient as the average difference in test scores between two districts that differ by 1 *SD* of average parental socioeconomic status. For each state, we compute both the gradient in Grade 3 and the average within-cohort change in the gradient, per grade, from Grades 3 to 8. Together, these two statistics provide information on the extent of between-district inequality of educational opportunity in elementary school and the extent to which inequality grows through the end of middle school. Second, we describe the bivariate distribution of these measures, noting the degree to which they vary and covary across states. Third, we examine how the two measures are associated with aspects of states’ education system inequity, economic inequality, and between-district racial and economic segregation in each state.

This study extends prior research on the socioeconomic achievement gap in several ways. First, using test score data from every public school district in the United States, it provides a detailed description of between-district socioeconomic achievement gradients in each state from Grades 3 to 8. This provides information not just on the degree of between-district inequality in each state but on when this inequality arises and how it grows across grade levels. Second, it provides suggestive evidence about the role that several key state characteristics may play in shaping between-district educational inequality. In doing so, it draws attention to structural features of state educational systems and patterns of segregation, and sheds light on the way that larger policy regimes and residential patterns are associated with educational inequality.

## Background and Conceptual Framework

We are interested here in the *between-district* association of average test performance and average socioeconomic status. To make this concrete, suppose we have two equally high- and two equally low-SES districts, one of each in both States A and B, and all four with the same racial/ethnic demographic makeup. We are interested in whether the achievement gap between the high- and low-SES districts in state A differs from that in State B, and if so, what state features might account for this difference. Note that our emphasis on the between-district SES–achievement association differs from the more commonly studied association between *individual* students’ academic performance and their family SES.

We consider several state characteristics that may exacerbate or reduce such between district differences in academic achievement: (1) inequality in school funding among school districts; (2) inequality in the distribution of effective teachers (proxied by measures of the proportion of novice and high-absence teachers) among districts; (3) inequality in children’s access to high-quality preschool environments; (4) between-district economic segregation; and (5) state-level economic inequality. Some of these are features of states’ preschool and K–12 education systems; others are features of states’ economic contexts. We discuss each of these state characteristics and their potential relationship to between-district academic achievement differences below.

### Inequality in School Funding

If higher school funding leads to higher academic outcomes, then states with more unequal funding should also, all else equal, have more unequal academic outcomes. Early, primarily correlational, research showed that per pupil school spending was largely uncorrelated with academic achievement (Hanushek, 2003). But recent studies using more rigorous quasi-experimental methods to estimate the effects of school funding consistently find that school funding positively affects students’ academic performance and graduation rates (see Jackson, 2018, for a review of this literature). Several studies focusing on the effect of school finance reforms (SFRs) designed to equalize funding among richer and poorer school districts have found that the reforms improved numerous educational outcomes, including test scores (LaFortune et al., 2018), educational attainment (Candelaria & Shores, 2015; Hyman, 2017), and wages (Jackson et al., 2016). Moreover, the impact of these reforms was often stronger among low-income families and in low-income districts (Jackson et al., 2016; Lafortune et al., 2018). These findings imply that additional state-level funding can play a significant role in increasing student achievement and that more equitable funding regimes may help equalize educational outcomes.

That said, school funding is not strongly correlated with school district socioeconomic status in most states. Prior to the 1970s, most states had highly unequal funding policies that relied partly on local property tax revenue to fund schools (Kozol, 1967, 1991). Because of the large differences in property values in different communities, schools in poor communities typically received far less funding than those in affluent communities. Beginning in the 1970s, however, several forces have led to greater within-state equity in school finance policies, including both court-mandated and legislatively initiated school finance reforms (SFRs) (Jackson et al., 2016). Since then, each state has legislated at least one finance reform intended to weaken the association between local property values and per pupil public education spending (Hoxby, 2001). As a result, school funding inequality is low in many states; this limited variation in funding inequality may make it difficult to observe any association between funding inequity and between-district achievement inequality.

### Unequal Access to Effective Teachers

A large body of research shows that teachers can have sizeable impacts on student outcomes but vary substantially in their effectiveness (Chetty, Friedman, & Rockoff, 2014; Goldhaber, 2007; Hanushek & Rivkin, 2010; Kane, Rockoff, & Staiger, 2008). Given this, we would expect that school districts with a larger proportion of effective teachers will have higher average test scores and larger average score growth rates, and that states where the most effective teachers are concentrated in affluent districts will have steeper between-district SES achievement gradients—and these gradients will grow faster from Grades 3 to 8—than in those where the average teacher effectiveness is uncorrelated with district socioeconomic conditions.

Teacher effectiveness measures are not broadly available or comparable, however. Instead, we use two proxy measures of teacher effectiveness: teacher experience and teacher absenteeism rates. Although teacher experience is only modestly correlated with teacher effectiveness after the first few years of a teacher’s career, most teachers become substantially more effective after their first few years of teaching (Papay & Kraft, 2015; Rockoff, 2004). We would expect greater between-district achievement disparities, then, in states in which novice teachers are concentrated in low-income districts. Likewise, teacher absenteeism has modest negative impacts on student learning (Clotfelter, Ladd, & Vigdor, 2009; Miller, Murnane, & Willett, 2008). Again, we would expect larger between-district SES achievement gradients and faster growth of the gradients from Grades 3 to 8 in states where teacher absenteeism rates are higher in lower SES districts.

### Unequal Access to High-Quality Preschool Programs

Access to high-quality preschool programs has long-term benefits for children, including improved educational outcomes as well as later adult outcomes and earnings (Heckman, Pinto, & Savelyev, 2013; Johnson & Jackson, 2017; Magnuson & Duncan, 2016). But preschool enrollment rates are lower, on average, among low-income students than higher income students. Indeed, one key argument in favor of expanding public preschool programs is that achievement gaps are partly driven by the lower preschool enrollment rates of low-income children (Magnuson, Meyers, & Waldfogel, 2007; Magnuson & Waldfogel, 2016). A second argument is that, because preschool programs are particularly effective for low-income children, expanding their access to preschool might substantially reduce achievement gaps (Burchinal, Vandergrift, Pianta, & Mashburn, 2010; Diamond, Barnett, Thomas, & Munro, 2007; Duncan & Sojourner, 2013; Elder & Lubotsky, 2009; Magnuson & Waldfogel, 2005; Wong et al., 2008). As a result of both of these factors, we might expect that states where the disparity between high- and low-income communities in access to high-quality preschool is small, later disparities in children’s academic outcomes will be small as well.

Two factors might work against this, however. First, there is considerable—though not universal—evidence that the academic effects of preschool enrollment fade by second or third grade (Barnett, 2011; Camilli, Vargas, Ryan, & Barnett, 2010; Hill, Gormley, & Adelstein, 2015; Magnuson & Duncan, 2016; Magnuson, Ruhm, & Waldfogel, 2007; Pianta, Barnett, Burchinal, & Thornburg, 2009). As a result, differences in preschool enrollment rates may have little discernable effect on disparities in academic achievement in the later elementary or middle school grades. Moreover, even if socioeconomic differences in preschool enrollment rates are associated with the between-district socioeconomic achievement gradient in elementary school, we do not expect that they will be related to the growth of the gradient after elementary school.

Second, equalizing preschool enrollment patterns does not necessarily mean that children in low- and high-income communities have access to preschool programs of equal quality. Indeed, Valentino (2018) finds that public preschool programs serving low-income, Black, and Hispanic students are of systematically lower quality on a number of dimensions than public preschool programs in the same state serving White and higher income children. Had Valentino’s study included data on private preschool programs as well, the quality disparities would likely have been even larger. Given the evidence that preschool effects tend to fade and that preschool quality is unevenly distributed, even within public preschool programs, greater equality in preschool enrollment patterns within a state may not necessarily lead to more between-district equality in educational outcomes, though that is the intended effect of policies designed to expand public preschool access.

### Between-District Economic Inequality and Segregation

Income inequality has been increasing in the United States for four decades (Piketty & Saez, 2003). When income inequality is high, the disparity in economic resources—as well as political power and social capital—between the richest and poorest families is large. This may lead to larger achievement gaps between children in high- and low-income families, given the large disparities in resources that families can deploy to provide educational opportunities for their children (Reardon, 2011b).

Income inequality also shapes the degree of economic segregation between school districts (Reardon & Bischoff, 2011). As income inequality has risen, so has economic segregation, particularly among families with children (Owens, 2016). Consequently, school districts have become increasingly economically segregated in recent decades (Owens et al., 2016). The growth of between-district income inequality and income segregation implies that both poverty and affluence may be highly concentrated in some districts. Because district boundaries partly determine students’ access to educational resources, this may shape disparities in students’ educational opportunities (McCoy, Connors, Morris, Yoshikawa, & Friedman-Krauss, 2015; Siegel-Hawley, 2013). For example, segregation may affect local labor markets; it may lead to greater sorting of teachers among school districts. In a state with little economic segregation among districts, teachers may be largely indifferent to where they teach, leading to only a weak association between teacher characteristics and school district socioeconomic status. But in a state with high segregation, teachers may regard the decision of where to teach as more salient, which would lead to greater sorting of teachers among districts and a potentially stronger association between district socioeconomic status and teacher effectiveness. Mechanisms like this might lead segregation to exacerbate between-district achievement gaps.

That said, it is not obvious that segregation will exacerbate the association between district socioeconomic status and achievement. If the average achievement in a school district is simply a function of that district’s characteristics and composition, then there is no reason to expect segregation to have any independent effect on district achievement or the gradient. Segregation certainly shapes the socioeconomic composition of a community and leads to greater variability in district average socioeconomic status, but that alone does not imply that the relationship between achievement and socioeconomic status would be stronger when segregation is higher. Thus, it is an open question whether segregation exacerbates the between-district socioeconomic achievement gradient.

## Research Questions and Hypotheses

In this article, we first estimate the between-district socioeconomic achievement gradient in third grade in each state and the rate at which each state’s gradient changes from third to eighth grade. Following a description of these gradients, we examine how state-level characteristics are associated with both (a) socioeconomic achievement gradients in Grade 3 and (b) the change in the socioeconomic achievement gradient across grades?

Although this study is primarily descriptive in nature, our discussion above suggests several hypotheses. First, we hypothesize that measures of between-district inequality and segregation—between-district income segregation and state-level income inequality—may be positively associated with between-district SES achievement gradients at Grade 3 and with the growth of the SES achievement gradient from Grades 3 to 8. The discussion above, however, leaves open the possibility that segregation may not be associated with the gradient or its growth.

Second, we expect that measures of state-level school system inequity—differences in preschool enrollment by income, differences in funding between high- and low-SES districts, and differences in the distribution of novice and high-absence teachers among high- and low-SES districts— may be positively associated with SES achievement gradients at Grade 3 and especially with the growth of the gradient from Grades 3 to 8. We do not, however, expect preschool enrollment patterns to be associated with the change in gradients from Grades 3 to 8, since any preschool effects on the gradient may be fully realized by Grade 3. Other measures of inequity in school resources, however, may have a continued effect and so would be associated with the growth of the gradient from Grades 3 to 8.

## Data and Models

### Data

We use test score data from the Stanford Education Data Archive (SEDA), which includes estimates of average test scores by district, subject, grade, and year for nearly all U.S. school districts (Fahle et al., 2018). The data include average scores on math and English/language arts (ELA) tests in Grades 3 to 8 from the 2008–2009 academic year through the 2015–2016 academic year. We standardize the SEDA average test score estimates within each grade–year–subject relative to the national student-level test score distribution; this scaling implies that all the estimates we produce are interpreted in grade–year–subject specific standard deviation units.^2^ For each school district, SEDA contains as many as 96 estimates (6 grades, 8 years, and 2 subjects) of average test scores. In some cases, estimates are missing because not all students in a given state–year–grade–subject took the same test; because some states did not administer standardized tests in 2014–2015; because of high opt out rates in some state–year–grades; and because of missingness in states’ reported test data in some state–grade–year–subjects (for detail, see Fahle et al., 2018). In total, we use 885,280 mean score estimates from 10,954 school districts (an average of 81 estimates per district) in 49 states.^3^

The SEDA data also include a measure of the average family SES in each school district in its covariate data set. This is a composite measure of SES, defined as the first principal component of the median income, the percent of adults with a bachelor’s degree or higher, the percentage of households with 5- to 17-year-olds in poverty, the SNAP (Supplemental Nutrition Assistance Program) receipt rate, the percentage of single mother–headed households, and the employment rate among adults aged 25 to 64 years in the district. We compute SES from American Community Survey (ACS) data from 2006 to 2010 and 2011 to 2015; then, average the two estimates to obtain a measure of average school district SES in the years 2006 to 2015. Based on sampling error information in the ACS, we construct standard errors for the SES measures; using these standard errors, we construct Empirical Bayes (EB) shrunken estimates of SES. We use these EB estimates of district SES in our regression models to obtain unbiased estimates of the SES achievement gradient in each state (because of measurement error in the SES variable, using the unshrunken SES values would lead to some modest attenuation of the gradients).

For state-level moderators of SES achievement gradients, we use four measures of state-level school system inequity. First is a measure of the difference in preschool enrollment between high- and low-income students. Ideally, we would like district-level preschool enrollment rates from which we could compute between-district enrollment gradients. But district-level preschool enrollment data are not available. Instead, we use ACS Public Use Microdata Sample data to estimate the difference in preschool enrollment rates between 4-year-olds in the highest and lowest income quintiles in the state. Higher values indicate higher inequity in early education access.

Second is a measure of between-district funding disparities. We regress district log per pupil revenue on school district SES. The coefficient on SES in this model describes the extent to which high-SES districts have more (or less) funding per pupil than low-SES districts. Again, higher values indicate greater inequity in school funding.

Third and fourth are two proxy measures of the distribution of effective teachers, one measuring the distribution of experienced teachers and one measuring the distribution of teacher absenteeism across districts. We obtain measures of the proportion of teachers with more than 2 years of experience and measures of the proportion of teachers who are absent less than 10 days/year from the Civil Rights Data Collection data. As above, we regress each of these on district SES to obtain coefficients that reflect the association between each proxy for teacher effectiveness and district SES. And as above, higher values indicate greater inequity in the distribution of experienced teachers and teachers with low absenteeism rates.

In addition, we include in the models two measures of state-level economic inequality: between-district income segregation and state-level income inequality. For segregation, we use 2006 to 2010 ACS data to compute the rank-order variance ratio index (*R*) of income segregation (Reardon, 2011a), using the method described by Reardon et al. (2018) to construct unbiased estimates of income segregation in each state. Higher value indicates higher between-district income segregation. To measure income inequality, we use the Gini coefficient, a measure of how unequally distributed household incomes are. We obtain state-level Gini coefficients from the ACS data for the years 2012 to 2016.

Finally, we construct a measure of the difference in average racial composition between high-SES and low-SES districts in each state. We use this measure as a control variable in our models, to eliminate confounds due to differences between states in their racial composition and in the association between race and SES. We construct this measure for each state by regressing the percentage of White residents in a district on district average SES. The slope of the regression line indicates the strength of the association between racial composition and SES in the state. A higher value on this variable indicates that White students are disproportionately concentrated in the highest SES districts in the state.

Table 1 includes descriptive statistics for the measures we use.

**Table 1:** Descriptive Statistics, District and State Sample Characteristics

Variable	*N*	*M*	*SD*	*Min*	*Max*
District level					
SES	10,954	0.20	0.91	−4.49	2.94
State level					
Q5 - Q1 preschool difference	49	0.20	0.07	0.01	0.30
Funding gradient	49	−0.03	0.05	−0.16	0.06
Experienced teacher gradient	49	0.01	0.02	−0.04	0.06
Teacher presence gradient	49	0.01	0.03	−0.12	0.12
Income segregation	49	0.08	0.05	0.00	0.19
Income inequality	49	0.46	0.02	0.42	0.51
Race–SES association	49	0.13	0.08	−0.02	0.30

### Models

Before describing the full model that we use to estimate the associations between state characteristics and SES gradients, we describe a simpler model to provide intuition into the model. Suppose we have a single cohort of students progressing through grades within a district. We can express their average test scores in grade *g* in school district *d* in state *s* as the sum of average scores in Grade 3 in district *d* plus a term representing the linear change in scores from Grades 3 to *g*:

1 $$Y_{gds}=\alpha_{0ds}+\alpha_{1ds}\left(g-3\right)+e_{gds},$$

where α_0*ds*_ is the average score in district *d* in Grade 3 and α_1*ds*_ is the linear rate of change in average test scores per grade within a given cohort of students in district *d*. Now suppose we express both α_0*ds*_ and α_1*ds*_ as (state-specific) linear functions of district *SES* :

2 $$\begin{array}{c} \alpha_{0ds}=\beta_{00s}^{*}+\beta_{01s}^{*}{SES}_{ds}+r_{0ds}^{*}\\ \alpha_{1ds}=\beta_{10s}^{*}+\beta_{11s}^{*}{SES}_{ds}+r_{1ds}^{*} \end{array}$$

We use the superscript * here to indicate that the parameters describe the bivariate associations between SES and α_0*ds*_ and α_1*ds*_. For our purposes, the key parameters of interest here are β^*^_01*s*_ (the association in state *s* between district SES and a district’s average Grade 3 achievement) and β_11s_^*^ (the association in state *s* between district SES and a district’s within-cohort, across-grade rate of change in test scores). Note that there are two equivalent ways to think about what β_11s_^*^ represents: It indicates how much faster or slower achievement increases within a cohort across grades in high-versus low-SES districts in state *s* ; equivalently, it indicates how the between-district SES achievement gradient in state *s* changes as students within a cohort progress through grades.

Next, suppose district average test scores are functions of both district SES and a vector of other district-level covariates (**X***_ds_*), which may be correlated with SES. Let ∂**X**_s_^*^ indicate the bivariate linear association between **X** and SES in state *s*. A positive value of ∂*X*_s_^*^ means that *X* is larger on average in high-SES districts in state *s* than in lower SES districts. Now, we include **X** in Equation (2) above:

3 $$\begin{array}{c} \alpha_{0ds}=\beta_{00s}+\beta_{01s}{SES}_{ds}+{\text{X}}_{ds}{\text{B}}_{0}+r_{0ds}\\ \alpha_{1ds}=\beta_{10s}+\beta_{11s}{SES}_{ds}+{\text{X}}_{ds}{\text{B}}_{0}+r_{0ds}. \end{array}$$

Next suppose that we can express the intercepts and *SES* gradients (the β_⋅⋅_*_s_*s in Equation (3) as linear functions of state characteristics (**W***_s_*):

4 $$\beta_{..s}=\gamma_{..0}+{\text{W}}_{s}\Gamma_{..}+u_{..s}$$

Now, substituting (4) into (3), we have

5 $$\begin{array}{ccc} \alpha_{0ds} & = & \gamma_{000}+{\text{W}}_{s}\Gamma_{00}+(\gamma_{010}+{\text{W}}_{s}\Gamma_{01}+u_{01s}){SES}_{ds}\\ & & +{\text{X}}_{ds}{\text{B}}_{0}+u_{000}+r_{0ds}\\ \alpha_{1ds} & = & \gamma_{100}+{\text{W}}_{s}\Gamma_{10}+(\gamma_{110}+{\text{W}}_{s}\Gamma_{11}+u_{11s}){SES}_{ds}\\ & & +{\text{X}}_{ds}{\text{B}}_{1}+u_{100}+r_{1ds.} \end{array}$$

Given the linearity in Equation (2), the partial derivative of (5) with respect to *SES* in state *s* is

6 $$\begin{array}{l} \frac{\partial \alpha_{0ds}}{\partial SES}{|}_{s}=\beta_{01s}^{*}=\gamma_{010}+{\text{W}}_{s}\Gamma_{01}+\partial {\text{X}}_{s}^{*}{\text{B}}_{0}+u_{01s}\\ \frac{\partial \alpha_{1ds}}{\partial SES}{|}_{s}=\beta_{11s}^{*}=\gamma_{110}+{\text{W}}_{s}\Gamma_{11}+\partial {\text{X}}_{s}^{*}{\text{B}}_{1}+u_{11s.} \end{array}$$

Equation (6) implies that both the state-specific *SES* gradient and the state-specific rate of change in the SES gradient as children progress through grades are functions of a set of state characteristics, including both measures of states’ between-district associations between district characteristics and *SES* (the ∂**X**_s_^*^ s) and other state characteristics (**W***_s_*). Moreover, the coefficient on ∂**X**_s_^*^ in these expressions is simply the within-state partial association between **X***_d_* and α_0*ds*_ or α_1*ds*_, controlling for *SES*. This implies that we can estimate both the **B** coefficients from Equation (3) and the Γ coefficients from Equation (4) by regressing states’ district-level SES achievement gradients on **W***_s_* and ∂**X**_s_^*^. This is useful if we cannot observe **X***_ds_*, but we can observe or estimate ∂**X**_s_^*^ from other sources.

In practice, we do not simply compute β^*^01*s* and β_11s_^*^ and then regress them on state characteristics as implied by Equation (6). Rather, we fit a multilevel model that combines Equations (1) to (3) above; in this model, grade–year– subject cells are nested in districts, which in turn are nested within states. Moreover, we pool data over two subjects and over multiple cohorts of students within a district. In addition, we incorporate the uncertainty in each district–grade– year–subject’s estimated average achievement level. Our general model is therefore:

7 Y^gybds=α0ds+α1ds(grade)+α2ds(cohort)+α3ds(math)+egybds+ϵgybdsα0ds=β00s+β01s(SESds)+r0dsα1ds=β10s+β11s(SESds)+r1dsα2ds=β20s+β21s(SESds)+r2dsα3ds=β30s+β31s(SESds)+r3dsβ00s=γ000+WsΓ000+u00sβ01s=γ010+WsΓ010+u01sβ10s=γ100+WsΓ100+u10sβ11s=γ110+WsΓ110+u11sβ20s=γ200β21s=γ210β30s=γ300β31s=γ310ϵgydbs~N(0,ωgybds2);egybds~(0,τ12);Rds~MVN(0,τ22);Us~MVN(0,τ32)

where Y^gybds is the estimated average test score for grade *g*, year *y*, subject *b*, district *d*, and state *s* (the standard error of this estimate is denoted ω_*gybds*_); *grade* is a continuous variable indicating the tested grade (centered at Grade 3); *cohort* is a continuous variable indicating the year students entered school (centered at *cohort* = 2007)^4^; *math* is an indicator equal to 0.5 if the tested subject is math and −0.5 if the subject is ELA. The centering of these variables implies that α_0*sd*_ is the average of the math and ELA test scores in district *d* in Grade 3 for the 2007 cohort. *SES_ds_* is the average SES composite for district *d* in state *s* ; **W***_s_* is a vector of grand-mean centered state-level covariates for state *s* (which may include ∂**X**_s_^*^, the between-district associations in state *s* between district covariates and SES described above). The *u*_*..s*_’s are multivariate normally distributed mean-zero state-level residuals with variance–covariance matrix τ^2^_3_ to be estimated; the *r_.sd_* s are multivariate normally distributed mean-zero district residuals with variance matrix τ_2_^2^ to be estimated; *e*_*gybds*_ is a normally distributed within-district (grade–year–subject) residual with mean zero and variance τ_1_^2^ to be estimated; and ε_*gybds*_ is a normally distributed mean-zero error with variance equal to ω^2^_*gybds*_, the (known) sampling variance of Y^gybds. Model estimation is performed using the HLM software (Raudenbush, Bryk, & Congdon, 2012).^5^

The key parameters of interest in the model are γ^010^, γ^110^, Γ_010_, and Γ_110_. The first two represent the average SES achievement gradient—the association between district SES and test score—at Grade 3 and its average growth per grade, respectively.^6^ The last two describe the associations between the vector **W** of state covariates and both the SES achievement gradient at Grade 3 and its linear change from Grades 3 to 8, respectively. In other words, they help us answer the questions: “How are state characteristics related to the association between district SES and district achievement in Grade 3?” and “How are these characteristics related to the change of this association as cohorts progress from Grades 3 to 8?”

## Results

**Figure 1:**
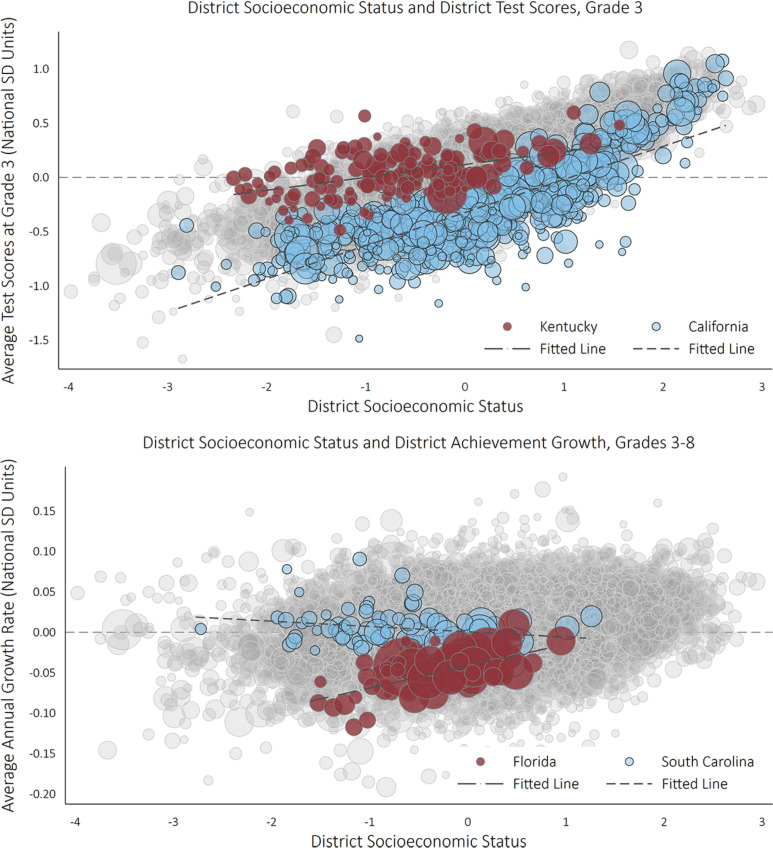
District mean achievement and achievement growth rate, by district socioeconomic status (SES).

Figure 1 describes overall association of district socioeconomic status with average test scores in Grade 3 (top figure) and with the average growth rate of test scores per grade in Grades 3 to 8 (bottom figure). Each gray bubble in the two plots represent one of the roughly 11,000 districts in the United States in our data. Overall, there is a strong positive (national) SES achievement gradient in Grade 3. The relationship between district SES and average test score growth is also positive, although the association is much weaker (see Reardon, 2019). The positive association between average test score growth and district SES implies that the (national) SES achievement gradient grows steeper from Grades 3 to 8 (because achievement grows more in high-than low-SES districts).

There is considerable variation in the strength of these bivariate relationships among states, however. The top panel of Figure 1, for example, highlights districts in Kentucky and California; the SES achievement gradient is much weaker in Kentucky than in California. Similarly, the relationship between district SES and test score growth is strong and positive in Florida, but flat in South Carolina (see bottom panel of Figure 1). This indicates that the SES achievement gradient grows steeper from Grades 3 to 8 in Florida but remains constant across grades in South Carolina. The large differences among states in the SES–achievement association and its growth across grades suggest that state-level factors may aggravate or ameliorate between-district inequality of educational opportunity.

**Figure 2:**
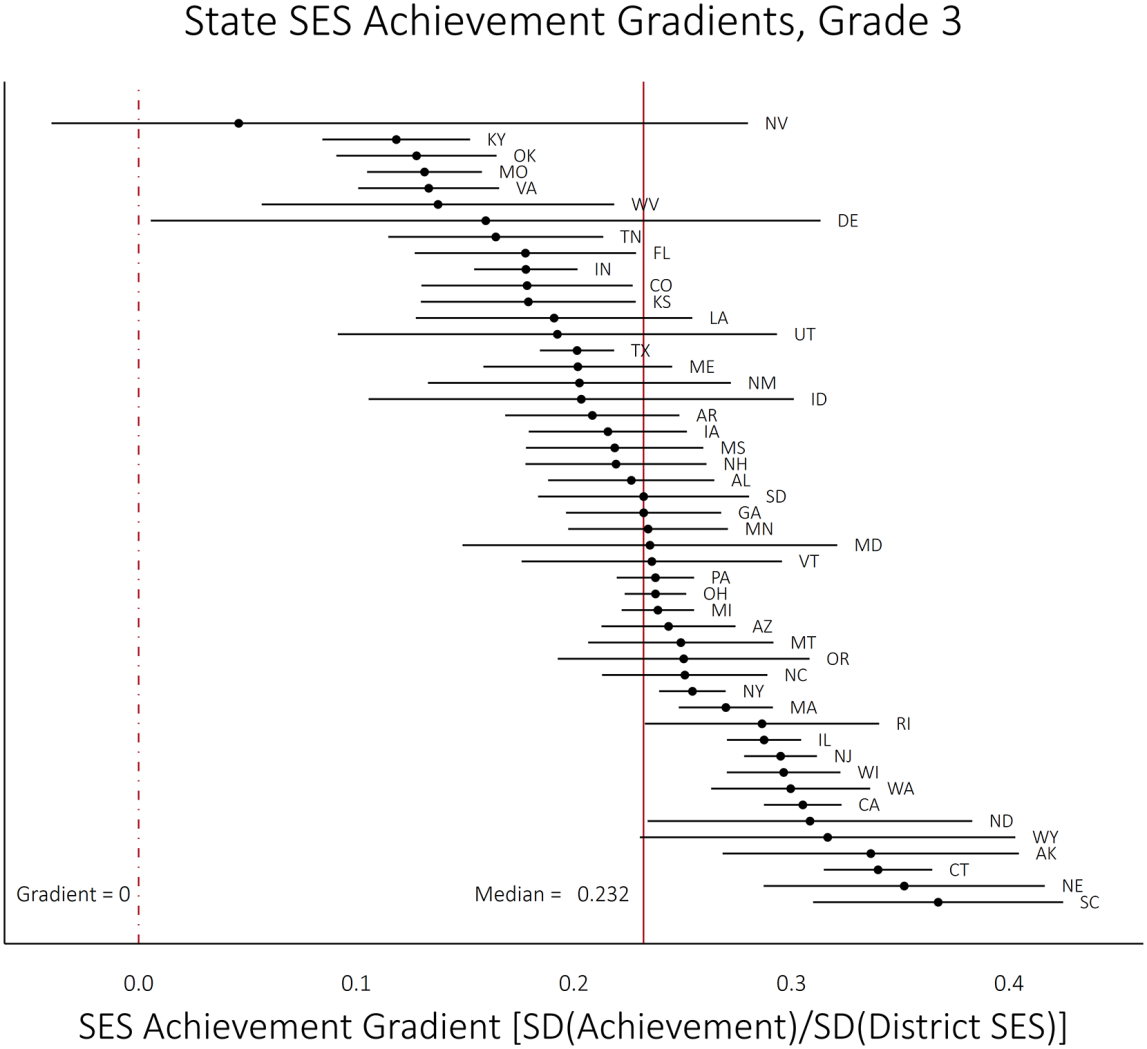
State socioeconomic status (SES) achievement gradients at Grade 3. *Note*. The lines represent the 95% confidence interval for each estimated gradient. The confidence interval for NV is truncated (its true lower bound is −0.19) for better display.

**Figure 3:**
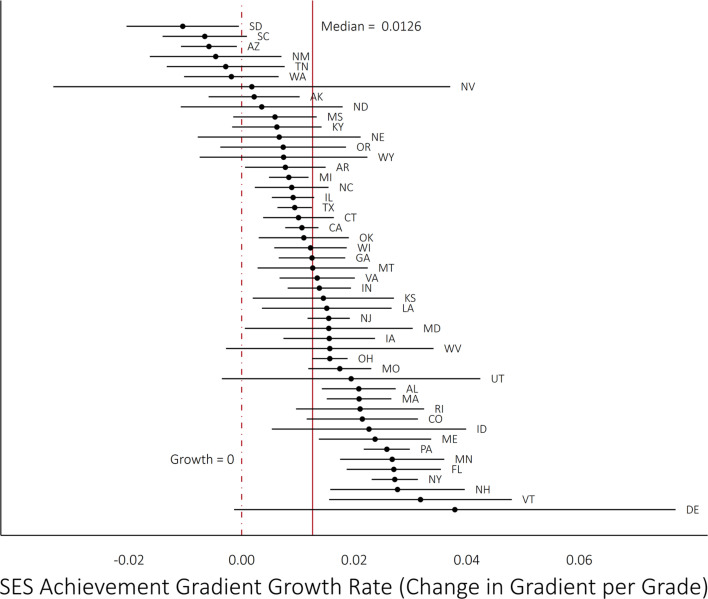
State socioeconomic status (SES) achievement gradient growth rates, Grades 3 to 8. *Note.* The lines represent the 95% confidence interval for each estimated gradient growth rate.

Figures 2 and 3 present the distribution of the SES gradients at Grade 3 and their growth per grade in each state.^7^ Each line represents the 95% confidence interval for the parameter, with the point estimate in the middle. State-specific point estimates of gradients and their growth are included in Appendix A. Figure 2 shows that the gradients have a median value of 0.232 in Grade 3: in the median state a 1 *SD* difference in district SES is associated with a difference in average test scores of 0.232 *SD*s (which corresponds, roughly, to two thirds of a grade level in test scores). South Carolina has the steepest estimated state-level SES achievement gradient (0.367), followed by Nebraska (0.352), Connecticut (0.340), and Alaska (0.336). Other than Nevada, whose gradient is very imprecisely estimated, Kentucky has the weakest association between district SES and test scores (0.118, less than one third as steep as in South Carolina), followed by Oklahoma (0.128) and Missouri (0.131). In no state (except for Nevada, whose gradient is very imprecisely estimated), does the 95% confidence interval on the Grade 3 gradient include 0.

One might wonder how the estimated SES achievement gradients shown in Figure 2 compare to estimates of the student-level achievement gap between high- and low-SES students. We do not have student-level family SES measures needed to compute this, but we can compute the achievement gap between poor and nonpoor students (as measured by free lunch eligibility) in each state in Grade 4 from NAEP data. This is not a perfect comparison, for several reasons: SES and free lunch eligibility measure somewhat different dimensions of socioeconomic conditions; the SES achievement gradient is measured in Grade 3, while the free lunch eligibility gap is measured in Grade 4; and both are measured with error (though both have reliabilities >.80). Nonetheless, the (measurement error corrected) correlation between the two measures is 0.49 (see Appendix B). This suggests that the district SES achievement gradient carries a considerable amount of information about the student-level association as well.

Figure 3 shows that in most—but not all—states, the SES achievement gradient grows from Grades 3 to 8. In about two thirds of states (32 out of 49 states), the estimated rate of change in the SES gradient is positive and its 95% confidence interval does not include 0; most of the other states have positive point estimates whose confidence interval includes 0, though six states have negative point estimates; for two of these states (South Dakota and Arizona), the point estimate is negative (−0.011 and −0.006, respectively) and the confidence interval does not include 0. The gradient grows the most rapidly in Delaware (0.038), Vermont (0.032), New Hampshire (0.028), New York (0.027), and Florida (0.027) (though Delaware’s growth rate is very imprecisely estimated).

**Figure 4:**
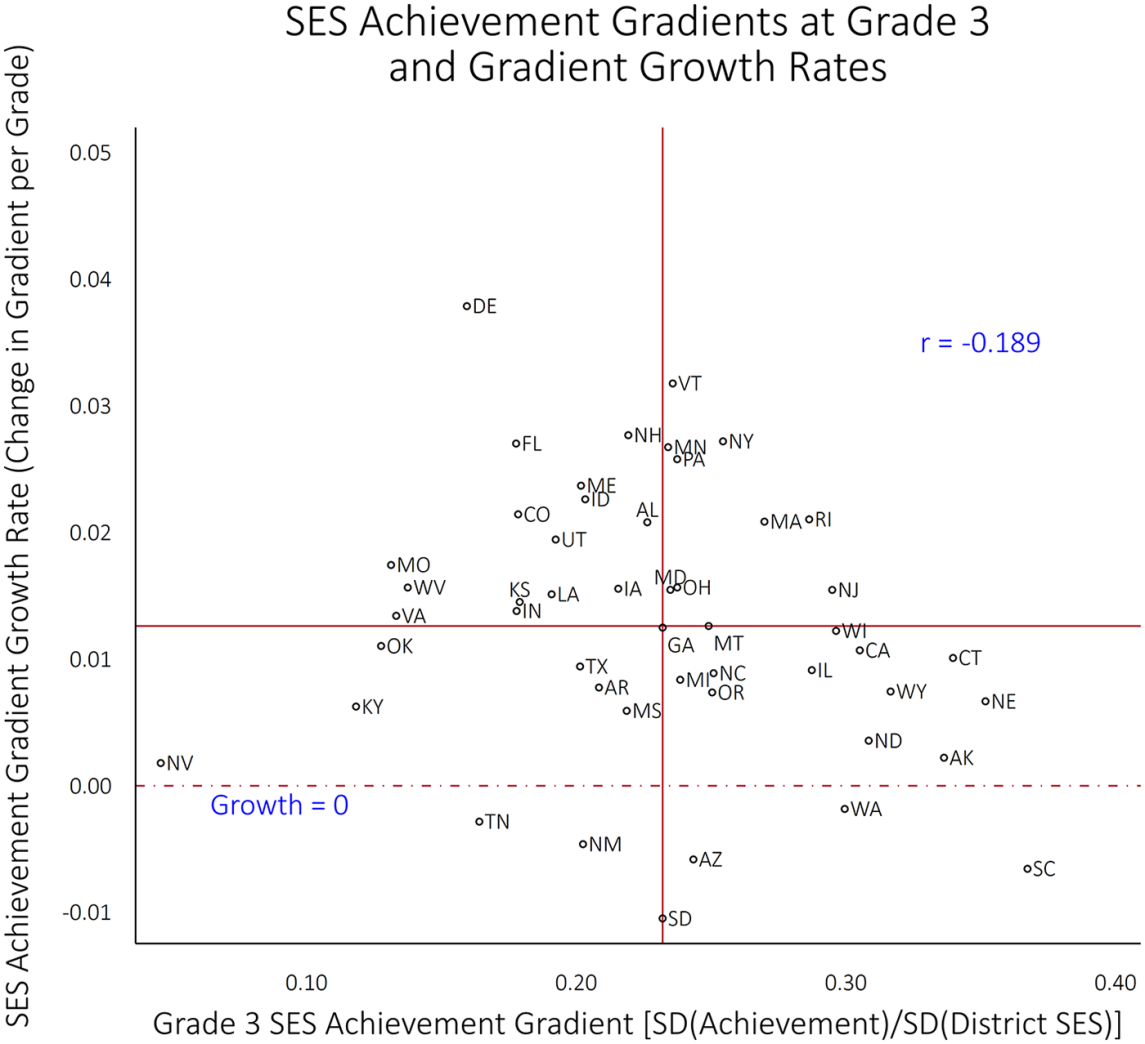
Socioeconomic status (SES) achievement gradients at Grade 3 and gradient growth rates.

Figure 4 plots the growth in SES achievement gradients from Grade 3 through Grade 8 against state-level SES achievement gradients at Grade 3; the red dashed lines— defined by the medians of the two measures—divide the figure into quadrants. States in the upper right quadrant (including VT, MA, NJ, NY, PA, RI, and MN; mostly wealthy states in the Northeast) have high SES achievement gradients in Grade 3 and above average growth rates from Grades 3 to 8, implying that they will have very high gradients in Grade 8. Conversely, those in the lower left (KY, TN, OK, NV, TX, AR, MS, and NM) have low Grade 3 gradients, and below average rates of change in their gradients (in some cases, negative) from Grades 3 to 8; these states will have the lowest SES achievement gradients in Grade 8. The other two quadrants include states with shallow initial gradients but above average growth rates (upper left) and states with steep initial gradients but slow growth rates (lower right). Note that the bivariate correlation (disattenuated to account for measurement error in both measures) between Grade 3 SES achievement gradients and the growth rate of these gradients is −0.189. That is, the extent to which states provide equal educational opportunities for children in high- and low-income districts during early childhood and elementary school is slightly negatively correlated with the extent to which they provide equalizing opportunities in the late elementary and middle school years.

**Figure 5:**
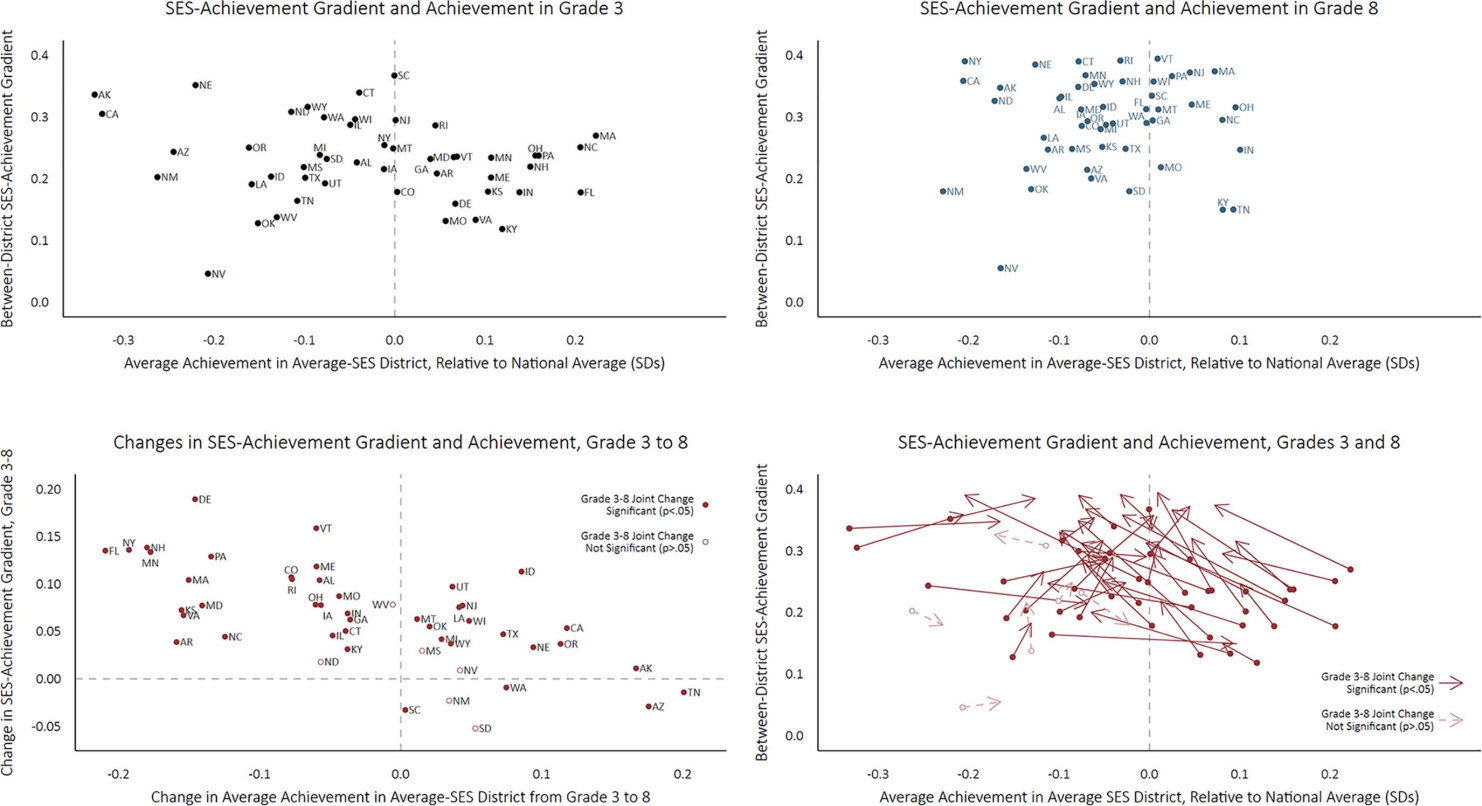
Change of achievement and socioeconomic status (SES) achievement gradient from Grades 3 to 8, by state.

Figure 5 shows how both average test scores and SES achievement gradients change in each state from Grades 3 to 8, with four panels highlighting different aspects of the patterns. The first (upper left) panel presents SES achievement gradients against average achievement in Grade 3. The second (upper right) panel plots these measures in Grade 8. The third (lower left) panel shows each state’s change in both the SES achievement gradient and average achievement from Grades 3 to 8. Here, dashed lines at 0 on each axis divide the figure into four quadrants. In the upper left quadrant are states where average achievement declines and the SES achievement gradient grows steeper from Grades 3 to 8— places where both equity and achievement declined across grades. In the lower right quadrant are states where both achievement and equity improve across grades.

The fourth panel displays the data from the top two panels in a single plot, with arrows describing each state’s movement in both the achievement and equity gradient dimensions from Grades 3 to 8. Arrows pointing right indicate an increase in average achievement from Grades 3 to 8; those pointing left indicate a decrease in average achievement in Grade 8 compared with Grade 3. Movement along the vertical axis reflects changes in the SES achievement gradient in the state. Arrows pointing upward reflect an increase in between-district inequality from Grades 3 to 8; those pointing downward indicate a decrease in between-district inequality. Note that the estimates used to produce Figure 5 are shown in Appendix A.

One of the striking patterns in Figure 5 is that only a handful of states (SC, NM, SD, WA, AZ, and TN) fall into the lower right quadrant in the third panel, moving in the direction of both increasing achievement and increasing between-district equality. In contrast, roughly half of the states fall into the opposite quadrant, exhibiting both declining achievement and equity. Indeed, there is a strong correlation between changes in achievement and changes in SES achievement gradients: States that have higher average growth rates tend to have lower rates of increase (or declines) in the SES achievement gradient (*r* =−0.747). In other words, states with the fastest average improvement in test scores from Grades 3 to 8 tend to have lower rates of growth (or even declines) in their between-district SES achievement gradient from Grades 3 to 8. This suggests that the goals of improving achievement and equity are not incompatible and may even reinforce one another.

That said, Figure 5 also shows that by eighth grade, only a few states (TN and KY, and to a lesser degree MO and IN) have both above average achievement and relatively low SES achievement gradients. The states with the most desirable achievement patterns in third grade (high achievement and low SES achievement gradients—those in the lower right part of the first panel) tend to move up and to the left in the figure by Grade 8, becoming both more unequal and lower achieving. Conversely, those with the opposite patterns in Grade 3 (low achievement and high SES achievement gradients) tend to move the right in the figure by eighth grade. The result is that both average achievement and SES achievement gradients vary less among states in Grade 8 than in Grade 3.

This pattern is reflected in Table 2, which reports the estimated (disattenuated) correlations among four state-level parameters of interest (the average achievement in Grade 3, the SES achievement gradient in Grade 3; the average annual growth rate in test scores from Grades 3 to 8, and the average annual change in the SES achievement gradient from Grades 3 to 8). States with higher average Grade 3 test scores tend to have lower SES achievement gradients in third grade (*r* = −0.419), but higher rates of increase in the SES achievement gradient from Grades 3 to 8 (*r* = 0.726) and slower rates of average achievement growth from Grades 3 to 8 (*r* = −0.789). In Figure 5, most of the states in the right side of the first panel (high-achieving states in Grade 3) fall into the upper right quadrant in the third panel, indicating lower average growth in test scores and increasing SES achievement gradients. Again, high achievement and low inequality are not incompatible, though the combination is rarer in Grade 8 than in Grade 3.

**Table 2:** Correlations and Standard Deviations, Achievement and Socioeconomic Status (SES) Achievement Gradient Parameters

	Grade 3 Achievement	Grade 3 SES Achievement Gradient	Grades 3–8 Achievement Growth	Grades 3–8 Growth in SES Achievement Gradient
Grade 3 achievement	0.132			
Grade 3 SES achievement gradient	−0.419	0.055		
Grades 3–8 achievement growth	−0.789	0.468	0.017	
Grades 3–8 growth in SES achievement gradient	0.726	−0.189	−0.747	0.007

*Note.* Diagonal terms indicate the standard deviation of the measure across states; off-diagonal terms indicate estimated correlations.

Tables 3 and 4 show how the SES achievement gradient and its average annual change from Grades 3 to 8 are associated with our covariates. Table 3 reports bivariate relationships of our covariates with SES gradients at Grade 3 and their growth across grades. Both the preschool enrollment difference and the funding gradient are weakly negatively associated (*r* = −0.287 and *r* = −0.266, respectively; *p* <.10) with the SES achievement gradient and weakly positively associated (*r* = 0.348 and *r* = 0.361, respectively; *p* < .05) with its growth per grade. This suggests that states where nonpoor kids have greater access to early education and school funding tend to have lower initial gradients but faster gradient growth across grades, on average. Segregation has a positive correlation with the average change in the SES achievement gradient from Grades 3 to 8 (*r* = 0.351; *p* < .05), implying that the gradients increase quicker in highly segregated places. The race-SES association is positively correlated with the SES achievement gradient (*r* = 0.419; *p* < .01), showing that states where white population is highly concentrated in higher SES districts have higher initial SES achievement gradients.

**Table 3:** Bivariate Correlations of State Covariates With SES Achievement Gradient and Gradient Growth

	Correlation with:	
	SES Gradient	SES Gradient Growth
Q5 - Q1 preschool difference	−0.287†	0.348*
Funding gradient	−0.266†	0.361*
Experienced teacher gradient	0.252	0.213
Teacher presence gradient	0.173	−0.260
Income segregation	0.163	0.351*
Income inequality	−0.086	0.165
Race–SES association	0.419**	−0.237

Note. SES = socioeconomic status.

† p < .1.

* p < .05.

** p < .01.

*** p < .001.

Table 4 reports the estimates from our hierarchical linear models. Model 1 includes no state-level covariates and describes the means and variances of the SES achievement gradients and their growth rates. The SES achievement gradient has a mean of 0.239 and a *SD* of 0.055 among states. The growth of the gradients has a mean of 0.010 and a *SD* of 0.007. These patterns of variation are reflected above in Figures 2 and 3.

**Table 4:** Hierarchical Linear Model Parameter Estimates

	Model 1		Model 2		Model 3		Model 4	
	*b*	*SE*	*b*	*SE*	*b*	*SE*	*b*	*SE*
Intercept								
Intercept	−0.024	0.019	−0.015	0.018	0.001	0.020	−0.002	0.019
Q5 - Q1 preschool difference			0.606	0.303*			0.608	0.293*
Funding gradient			0.140	0.455			−0.327	0.520
Experienced teacher gradient			−0.205	0.946			−0.036	0.931
Teacher presence gradient			0.259	0.557			0.182	0.540
Income segregation					0.642	0.419	0.546	0.410
Income inequality					1.024	1.031	1.042	1.151
Race–SES association			−0.298	0.239	−0.469	0.212*	−0.390	0.242
SES slope	0.239	0.009***	0.235	0.008***	0.240	0.009***	0.240	0.008***
Q5 - Q1 preschool difference			−0.115	0.138			−0.134	0.131
Funding gradient			−0.051	0.207			−0.161	0.238
Experienced teacher gradient			0.783	0.481			0.780	0.476
Teacher presence gradient			0.170	0.298			0.157	0.285
Income segregation					0.396	0.191*	0.437	0.187*
Income inequality					−0.688	0.468	−0.236	0.540
Race–SES association			0.205	0.113†	0.317	0.099**	0.208	0.115†
Grade								
Intercept	−0.004	0.002	−0.004	0.002†	−0.006	0.003*	−0.005	0.003*
Q5 - Q1 preschool difference			−0.064	0.042			−0.067	0.041
Funding gradient			−0.017	0.063			0.032	0.073
Experienced teacher gradient			−0.001	0.132			−0.029	0.133
Teacher presence gradient			−0.044	0.078			−0.042	0.077
Income segregation					−0.035	0.058	−0.023	0.058
Income inequality					−0.149	0.144	−0.163	0.163
Race–SES association			0.018	0.033	0.035	0.030	0.030	0.034
SES slope	0.010	0.001***	0.011	0.001***	0.012	0.001***	0.011	0.001***
Q5 - Q1 preschool difference			0.017	0.018			0.015	0.018
Funding gradient			0.023	0.026			0.015	0.032
Experienced teacher gradient			0.055	0.065			0.047	0.067
Teacher presence gradient			−0.024	0.041			−0.031	0.040
Income segregation					0.046	0.025†	0.038	0.025
Income inequality					0.009	0.063	−0.018	0.073
Race–SES association			−0.007	0.015	−0.015	0.014	−0.006	0.016
Math								
Intercept	−0.012	0.001***	−0.012	0.001***	−0.012	0.001***	−0.012	0.001***
SES slope	0.004	0.001**	0.004	0.001**	0.004	0.001**	0.004	0.001**
Cohort								
Intercept	−0.003	0.000***	−0.003	0.000***	−0.003	0.000***	−0.003	0.000***
SES slope	0.003	0.000***	0.003	0.000***	0.003	0.000***	0.003	0.000***
Number of observations	885,280		885,280		885,280		885,280
Number of districts		10,954		10,954		10,954		10,954
Number of states		49		49		49		49
Within-district SD		0.116		0.116		0.116		0.116
Within-state intercept SD		0.188		0.188		0.188		0.188
Within-state grade slope SD		0.038		0.038		0.038		0.038
Within-state math slope SD		0.122		0.122		0.122		0.122
Within-state cohort slope SD		0.026		0.026		0.026		0.026
Between-state intercept SD		0.132		0.118		0.119		0.113
Between-state SES gradient SD		0.055		0.050		0.049		0.046
Between-state grade slope SD		0.017		0.016		0.016		0.016
Between-state grade * SES SD		0.007		0.006		0.006		0.006
Percentage of variance explained								
Between-state SES gradient variance				18.6%		21.1%		29.2%
Between-state grade * SES variance				20.0%		16.7%		25.0%

*Note.* SE = standard error; SES = socioeconomic status.

† p < .10.

* p < .05.

** p < .01.

*** p < .001.

Models 2 and 3 in Table 4 each include different sets of covariates. The measures of between-district inequality (Model 3) explain roughly the same amount of the variance in SES gradients and their growth rates as do the measures of school system inequity (Model 2): each model explains 17% to 29% of the variance in both SES achievement gradients and the rate of change of the gradients. The model containing all the measures (Model 4) explains 29% and 25% of the variances, respectively.

The coefficient estimates from Model 4 describe the partial associations of the state covariates with the SES achievement gradient and its growth. Notably, between-district income segregation is the only state characteristic that is positively associated (*p* <.05) with the SES achievement gradient at Grade 3 after controlling for all the other state covariates in the model. In a state with income segregation 1 *SD* (0.05) above average, the SES achievement gradient is predicted to be 0.022 (= 0.437 × 0.05) steeper than average, approximately 9% larger (= 0.022 / 0.239) than the average gradient.

The race–SES association also has a marginally significant positive relationship (*p* <.10) with SES achievement gradients, indicating that district SES and average test scores are modestly more strongly associated with one another in states where district SES is highly correlated with racial composition. Specifically, in a state with a race–SES association 1 *SD* (0.09) higher than average, the SES achievement gradient is predicted to be 7% (=0.208 × 0.08) / 0.239) larger than the average gradient.

In contrast to the between-district inequality variables, none of the measures of school system inequity are significantly associated with either the SES achievement gradient or its growth from Grades 3 to 8 in the full model.^8^

## Discussion

Do states vary in the extent to which differences between districts in socioeconomic status are associated with differences between districts in educational outcomes? Or is the pattern of association between socioeconomic context and academic outcomes stable across states? Our analysis suggests that state contexts do play a role in shaping the socioeconomic equality of educational outcomes, though the mechanisms are not completely clear from our analyses.

We find substantial variation among states in the SES achievement gradient, and in its growth across grades. The gradient is three times steeper in the states with the strongest association between district SES and average achievement than in those with the weakest association. Moreover, the degree to which the gradients change from Grades 3 to 8 varies considerably. In some states, there is no significant change in gradient; in others—such as Delaware, Vermont, Florida, New York, and New Hampshire—the gradient nearly doubles in 5 years. These patterns suggest that state-level features of the educational system shape the relationship between educational opportunity and socioeconomic context. This finding is important because it suggests state policies may be an important lever for improving educational equity.

Not only do some state contexts appear to produce more equitable patterns of achievement but our results indicate it is possible—though uncommon—for states to improve achievement at the same time as they temper the association between socioeconomic conditions and achievement. In third grade, there are several states where achievement is high and the SES achievement gradient is low. And states where the SES achievement declines or remains stable from Grades 3 to 8 generally have increasing achievement at the same time. Tennessee and Kentucky, in particular, have both very high eighth grade achievement in their average SES districts and among the very weakest SES achievement gradients in the United States they serve as existence proofs that the goals of equity and high achievement are compatible.

Our study does not, however, identify specific causes or policy levers that might be used to reduce inequality. Three specific policy levers—recruiting experienced, lower absent teachers to low-income districts, providing additional funding to low-income districts, and expanding access/enrollment in preschool for low-income students—have been widely suggested as ways of reducing socioeconomic disparities in educational outcomes. We do not find evidence, however, that these factors are associated with the strength of the SES achievement gradient. To be clear, this does not mean these factors do not matter or might not be effective. Our study does not provide causal estimates of the effects of these state policies; it merely says that there is no clear correlational association between funding regimes, preschool enrollment patterns, or teacher experience and absenteeism patterns and the between-district SES achievement gradient. Other studies with a strong causal warrant provide much clearer evidence about the effects of their effects.

We do find that SES achievement gradients are steeper in states that are more economically segregated. This finding is in some ways quite striking. It suggests that economic segregation is associated with SES achievement gaps in some ways other than simply through the way it shapes local economic context. The finding that segregation is associated with steeper SES achievement gradients is particularly troubling given recent trends of increasing economic segregation (Reardon et al., 2018).

It is not exactly clear, however, what mechanisms might drive this association. One possibility is that there are nonlinear effects of socioeconomic context on educational opportunity, so that educational outcomes are not particularly sensitive to small differences in district context for districts with near-average SES, but that the effects of extreme socioeconomic advantage and extreme disadvantage are both very strong. This might be the case if there are threshold effects or tipping point effects, so that there is a point where community disadvantage becomes so extreme that it dramatically limits all students’ opportunities, or a point where advantage becomes so extreme that it expands all students’ educational success. This would manifest itself as a nonlinear pattern of association between average achievement and average SES, but such a pattern is not evident in the data (see Figure 1).

Another possibility is that segregation patterns are correlated with the unequal distribution of some other educational resource. For example, it may be that teacher labor markets operate differently under high levels of segregation than low segregation, leading to much more between-district sorting of teachers in highly segregated states, in ways that are not captured by our measures of teacher experience and absenteeism gradients. Teachers may be relatively indifferent to where they teach when districts differ little in economic or racial composition, but more sensitive to location when districts differ substantially. If so, the correlation of district SES and teacher quality may be more pronounced in highly segregated states than in less segregated states. Of course, this is not the only possible explanation or mechanism but it is one type of mechanism that would explain the strong association we find between segregation and the strength of the SES achievement gradient. Other explanations might involve outsize between-district differences in political power under conditions of high economic segregation or differences in public and private noneducational investments in rich and poor districts under conditions of high segregation.

Although the descriptive aspects of this study clearly show substantial variation among states in their between-district socioeconomic achievement gradients, the correlational aspects of the study have several important limitations. First, some of the measures do not capture key features of state policies and contexts. For example, the measure of preschool enrollment differences is noisy (because it is based on sample data) and does not capture differences in preschool quality. The measure of funding does not capture differences in how funds are used. The measures of teacher effectiveness are weak proxies for true effectiveness. Second, the study is primarily descriptive and exploratory; our design does not provide a warrant for causal interpretations of the correlations we report. Third, our ability to statistical control for potential state-level confounders is limited by the small number of degrees of freedom in the study. With only 49 states in the study, we are limited in the number of variables we can control for in the multivariate models.

Nonetheless, the study does provide clear evidence that the association between local socioeconomic conditions and educational opportunities differs considerably across states. This finding suggests that state policies or state contexts may exacerbate or ameliorate educational inequality; they may do so partly by shaping levels of between-district segregation. Future research might fruitfully examine a wider range of state policies—including health care policies, child care funding policies, and other nonschooling policies—to determine what strategic policy interventions might lead to more equal educational opportunities. Rather than expect each local school or district to address the challenges of inequality, particularly in highly segregated states, we might more reasonably ask states to play a larger role.

## Figures and Tables

**Figure:**
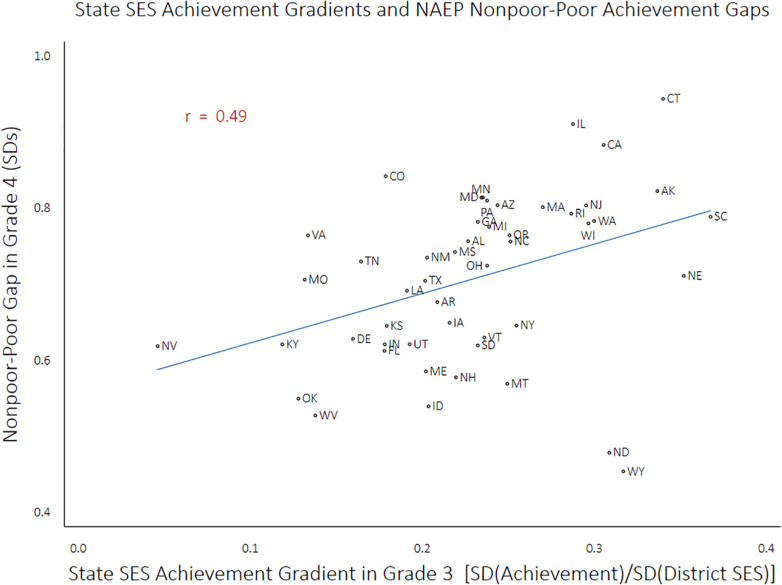
Socioeconomic status (SES) achievement gradients compared with student-level nonpoor–poor achievement gaps *Note*. Nonpoor–poor gap is the gap between non–free lunch eligible and free lunch eligible students, as measured using NAEP data. The correlation is disattenuated to account for measurement error in both measures.

## Notes

1 Note that although Hanushek et al. (2019) conclude that the socioeconomic achievement gap has been relatively constant over the past few decades, three of the four studies they rely on indicate the gap grew in the past few decades; one shows it declined.

2 One implication of this scaling is that the changes in gradients we estimate are interpreted relative to the national grade-specific student-level score distribution, rather than relative to an absolute performance metric.

3 Washington D.C. and Hawaii are excluded from the study because they each contain only a single school district.

4 Specifically, *cohort* refers to the spring of the kindergarten year. So, for example, *cohort* = 2007 indicates the middle cohort in our data, whom we observe in third grade in 2010, fourth grade in 2011, and so on, through eighth grade in 2015.

5 An alternate formulation of the model would treat the *u*_*..s*_ as fixed, rather than random. This would be appropriate, given that the data include the full set of states (except for HI and DC, which have only a single district). However, such a model exceeds the parameter limits of the HLM software. In lieu of fitting Model 7 with fixed effects at the state level, we instead fit a two-level model to each state’s data separately:

Y^gybds=α0ds+α1ds(grade)+α2ds(cohort)+α3ds(math)+egybds+ϵgybdsα0ds=β00s+β01s(SESds)+r0dsα1ds=β10s+β11s(SESds)+r1dsα2ds=β20s+β21s(SESds)+r2dsα3ds=β30s+β31s(SESds)+r3dsϵgydbs~N(0,ωgybds2);egybds          ~N(0,τ12);Rds~MVN(0,τ22)

From this, we obtain B^ds, the vector of estimated β*_..s_* ’s for each state, and its covariance matrix. We use B^ds in the descriptive figures below. To obtain estimates of Г_010_ and Г_110_ shown below in Tables 3 and 4, however, we fit the state random effects Model 7 above.

6 Note that because the test scores are scaled in terms of grade-and year-specific national student-level standard deviations, the growth of the gradient is measured relative to the grade-specific national student score distribution. If the true distribution of scores narrows or increases in later grades, as is evident in some vertically scaled assessments (Bloom, Hill, Black, & Lipsey, 2008; Dadey & Briggs, 2012), then the changes in gradients would be smaller or larger, respectively, than those we estimate here.

7 The estimates here are based on the model described in Note 5 above; that is, these are the fixed effects estimates from each state, rather than the EB estimates from the model in Equation (7).

8 In Appendix C, we conduct a set of sensitivity analyses to determine whether the results shown in Model 4 are robust to the removal of influential outlier districts and to the inclusion of time-varying SES measures. We find that the results are substantively unchanged by these changes to the sample and model.

## Appendix A

**Table:** Achievement and SES Achievement Gradient Parameter Estimates, by State

State	Grade 3 Achievement	Grade 3 SES Gradient	Grade 8 Achievement	Grade 8 SES Gradient	Achievement Growth	SES Gradient Growth
AK	−0.3319	0.3364	−0.1652	0.3475	0.0333	0.0022
AL	−0.0420	0.2264	−0.0995	0.3304	−0.0115	0.0208
AR	0.0465	0.2084	−0.1123	0.2473	−0.0318	0.0078
AZ	−0.2446	0.2435	−0.0689	0.2144	0.0351	−0.0058
CA	−0.3236	0.3052	−0.2059	0.3587	0.0235	0.0107
CO	0.0027	0.1785	−0.0749	0.2856	−0.0155	0.0214
CT	−0.0393	0.3397	−0.0784	0.3902	−0.0078	0.0101
DE	0.0670	0.1595	−0.0788	0.3488	−0.0291	0.0379
FL	0.2056	0.1777	−0.0037	0.3129	−0.0419	0.0270
GA	0.0393	0.2320	0.0035	0.2945	−0.0072	0.0125
IA	−0.0120	0.2156	−0.0685	0.2934	−0.0113	0.0156
ID	−0.1369	0.2034	−0.0513	0.3165	0.0171	0.0226
IL	−0.0492	0.2874	−0.0975	0.3330	−0.0097	0.0091
IN	0.1379	0.1779	0.1003	0.2469	−0.0075	0.0138
KS	0.1031	0.1790	−0.0523	0.2516	−0.0311	0.0145
KY	0.1188	0.1184	0.0811	0.1496	−0.0075	0.0063
LA	−0.1582	0.1909	−0.1169	0.2665	0.0083	0.0151
MA	0.2224	0.2698	0.0722	0.3741	−0.0301	0.0209
MD	0.0651	0.2349	−0.0756	0.3123	−0.0282	0.0155
ME	0.1065	0.2018	0.0467	0.3203	−0.0120	0.0237
MI	−0.0829	0.2386	−0.0541	0.2804	0.0058	0.0084
MN	0.1066	0.2341	−0.0707	0.3678	−0.0355	0.0267
MO	0.0562	0.1313	0.0126	0.2185	−0.0087	0.0174
MS	−0.1007	0.2187	−0.0856	0.2483	0.0030	0.0059
MT	−0.0018	0.2491	0.0097	0.3122	0.0023	0.0126
NC	0.2052	0.2510	0.0807	0.2954	−0.0249	0.0089
ND	−0.1145	0.3085	−0.1713	0.3263	−0.0113	0.0036
NE	−0.2203	0.3518	−0.1264	0.3851	0.0188	0.0067
NH	0.1499	0.2193	−0.0298	0.3578	−0.0360	0.0277
NJ	0.0009	0.2950	0.0447	0.3723	0.0088	0.0155
NM	−0.2625	0.2025	−0.2283	0.1794	0.0068	−0.0046
NV	−0.2066	0.0459	−0.1647	0.0549	0.0084	0.0018
NY	−0.0116	0.2544	−0.2041	0.3905	−0.0385	0.0272
OH	0.1555	0.2375	0.0952	0.3158	−0.0121	0.0157
OK	−0.1513	0.1277	−0.1309	0.1829	0.0041	0.0110
OR	−0.1612	0.2504	−0.0481	0.2873	0.0226	0.0074
PA	0.1593	0.2374	0.0249	0.3664	−0.0269	0.0258
RI	0.0448	0.2864	−0.0322	0.3916	−0.0154	0.0210
SC	−0.0005	0.3674	0.0027	0.3346	0.0006	−0.0066
SD	−0.0752	0.2320	−0.0223	0.1796	0.0106	−0.0105
TN	−0.1078	0.1641	0.0927	0.1499	0.0401	−0.0028
TX	−0.0991	0.2014	−0.0265	0.2485	0.0145	0.0094
UT	−0.0771	0.1924	−0.0405	0.2896	0.0073	0.0194
VA	0.0894	0.1333	−0.0645	0.2004	−0.0308	0.0134
VT	0.0688	0.2358	0.0089	0.3947	−0.0120	0.0318
WA	−0.0782	0.2996	−0.0034	0.2904	0.0150	−0.0018
WI	−0.0439	0.2964	0.0045	0.3575	0.0097	0.0122
WV	−0.1305	0.1375	−0.1362	0.2158	−0.0011	0.0157
WY	−0.0964	0.3166	−0.0609	0.3538	0.0071	0.0074
Min	−0.3319	0.0459	−0.2283	0.0549	−0.0419	−0.0105
Max	0.2224	0.3674	0.1003	0.3947	0.0401	0.0379

## Appendix C

### Sensitivity Analyses

We conduct two sets of analyses to assess how sensitive our results are to different ways of estimating the SES gradients in each state. The first analyses remove high-influence districts from the analyses to ensure that our results are not driven by a few high-leverage outlier districts. The second analyses using a time-varying measure of district SES to take into account changes in SES over time within districts.

To identify high-influence districts, we first fit an ordinary least squares regression of district-level average test scores (based on pooling data in each district across grades, years, and subject) on district SES for each state. We compute an influence statistic, DFBETA,^9^ for each district. We then remove high-influence districts from the data and refit Model 4 of Table 4. We use two different approaches to identifying high-influence districts. The first approach defines a district as high-influence if DFBETA > $2/\sqrt{n}$ (where *n* is the number of districts in the state), as suggested by Belsley, Kuh, and Welsch (1980). The second approach simply removes from each state the *x* percent of districts with the highest absolute values of DFBETA. For this approach, we use values of *x* ∈ {1, 2,…,5}.

To take into account within-district changes in average SES, we construct a time-varying measure of SES from ACS data. For 2009, we use data from the 2005 to 2009 ACS (so that the 2009 measure of district SES represents the average SES of children in the district over the last 5 years); for 2010, we use data from the 2006 to 2010 ACS; and so on. We then fit a modified version of Model 4 from Table 4 that includes this time-varying SES measure:

A1 Y^gybds=α0ds+α1ds(grade)+α2ds(cohort)+α3ds(math)+egybds+ϵgybdsα0ds=β00s+β01s(SES¯ds)+r0dsα1ds=β10s+β11s(SES¯ds)+r1dsα2ds=β20s+β21s(SES¯ds)+r2dsα3ds=β30s+β31s(SES¯ds)+r3dsα4ds=β40sβ00s=γ000+WsΓ000+u00sβ01s=γ010+WsΓ010+u01sβ10s=γ100+WsΓ100+u10sβ11s=γ110+WsΓ110+u11sβ20s=γ200β21s=γ210β30s=γ300β31s=γ310ϵgydbs~N(0,ωgybds2);egybds~(0,τ12);         ~MVN(0,τ22);Us~MVN(0,τ32)

where *SESC* is the district-centered measure of time-varying SES and ${\overset{¯}{SES}}_{ds}$ is the average socioeconomic status composite for district *d* in state *s* over the years.

Table in Appendix C shows that the primary results are substantively unchanged by the removal of high-influence districts and the use of time-varying SES. The average SES achievement gradient is roughly 0.25, and the average growth rate of the gradient is roughly 0.12, regardless of the data and mode. In each model, between-district income segregation is significantly associated with the SES achievement gradient; indeed the association is stronger when high-influence districts are removed, suggesting that the estimates in Table 4 may be somewhat conservative. Likewise, when high-influence districts are removed the experienced teacher gradient is more strongly associated with the SES achievement gradient, though this association is significant in only several of the models. In general, the sensitivity analyses suggest that our results are robust to the removal of high-influence districts and the inclusion of time-varying SES measures.

**Table:** Sensitivity Analyses

	Using Full Sample (see Table 4)	Models Dropping Influential Districts						Models Using Time-Varying SES
		1% Dropped	2% Dropped	3% Dropped	4% Dropped	5% Dropped	Cutoff Dropped
Intercept								
SES slope	0.240 (0.008)***	0.240 (0.008)***	0.239 (0.009)***	0.240 (0.009)***	0.239 (0.009)***	0.238 (0.009)***	0.239 (0.009)***	0.248 (0.009)***
Q5 - Q1 preschool	−0.134 (0.131)	−0.103 (0.133)	−0.050 (0.136)	−0.058 (0.137)	−0.048 (0.138)	−0.076 (0.139)	−0.036 (0.141)	−0.199 (0.138)
difference								
Funding gradient	−0.161 (0.238)	−0.242 (0.243)	−0.244 (0.248)	−0.246 (0.249)	−0.293 (0.252)	−0.326 (0.253)	−0.257 (0.256)	−0.195 (0.250)
Experienced Teacher	0.780 (0.476)	0.896 (0.483)†	0.984 (0.493)*	0.883 (0.497)†	0.939 (0.508)†	0.954 (0.511)†	1.251 (0.517)*	0.707 (0.502)
Gradient								
Teacher presence	0.157 (0.285)	0.128 (0.290)	0.015 (0.297)	0.012 (0.298)	0.042 (0.311)	−0.001 (0.312)	−0.094 (0.321)	0.156 (0.301)
gradient								
Income segregation	0.437 (0.187)*	0.599 (0.191)**	0.630 (0.195)**	0.635 (0.196)**	0.672 (0.197)**	0.694 (0.198)**	0.589 (0.201)**	0.392 (0.197)*
Income inequality	−0.236 (0.540)	−0.129 (0.551)	−0.112 (0.563)	−0.071 (0.566)	−0.021 (0.573)	−0.008 (0.576)	0.210 (0.585)	−0.212 (0.569)
Race–SES	0.208 (0.115)†	0.164 (0.117)	0.171 (0.120)	0.203 (0.121)†	0.211 (0.122)†	0.193 (0.123)	0.131 (0.125)	0.204 (0.121)†
association								
Grade								
SES Slope	0.011 (0.001)***	0.011 (0.001)***	0.011 (0.001)***	0.011 (0.001)***	0.010 (0.001)***	0.010 (0.001)***	0.010 (0.001)***	0.012 (0.001)***
Q5 - Q1 preschool	0.015 (0.018)	0.015 (0.017)	0.016 (0.018)	0.016 (0.019)	0.021 (0.019)	0.017 (0.018)	0.021 (0.018)	0.019 (0.018)
difference								
Funding gradient	0.015 (0.032)	0.015 (0.032)	0.013 (0.033)	0.007 (0.034)	0.004 (0.035)	0.001 (0.033)	−0.006 (0.033)	0.017 (0.032)
Experienced Teacher	0.047 (0.067)	0.048 (0.067)	0.048 (0.068)	0.049 (0.071)	0.055 (0.073)	0.061 (0.070)	0.023 (0.071)	0.059 (0.069)
Gradient								
Teacher presence	−0.031 (0.040)	−0.034 (0.040)	−0.038 (0.042)	−0.044 (0.043)	−0.051 (0.045)	−0.049 (0.044)	−0.057 (0.045)	−0.037 (0.042)
gradient								
Income segregation	0.038 (0.025)	0.034 (0.025)	0.034 (0.025)	0.033 (0.026)	0.032 (0.027)	0.025 (0.025)	0.033 (0.026)	0.035 (0.025)
Income inequality	−0.018 (0.073)	0.006 (0.073)	0.013 (0.075)	0.026 (0.077)	0.039 (0.080)	0.052 (0.076)	0.050 (0.076)	−0.020 (0.075)
Race–SES	−0.006 (0.016)	−0.008 (0.016)	−0.010 (0.016)	−0.011 (0.017)	−0.014 (0.017)	−0.009 (0.016)	−0.007 (0.016)	−0.006 (0.016)
association								
Number of observations	885,280	876,277	867,068	858,300	849,370	840,228	825,647	766,093
Number of districts	10,954	10,846	10,735	10,626	10,515	10,405	10,224	10,935
Number of states	49	49	49	49	49	49	49	49

Note. DFBETA cutoff is set to be as $2/\sqrt{n}$, following Belsley et al. (1980). SES = socioeconomic status.

†p < .10. *p < .05. **p < .01. ***p < .001.

## Authors

HEEWON JANG is a PhD student in the Graduate School of Education at Stanford University. Her research interests include segregation, educational inequality, achievement gaps, and educational policy.

SEAN F. REARDON is the endowed professor of poverty and inequality in education and is professor (by courtesy) of sociology at Stanford University. His research focuses on the causes, patterns, trends, and consequences of social and educational inequality, the effects of educational policy on educational and social inequality, and in applied statistical methods for educational research.

## References

[cit0001] Barnett W. S. (2011). Effectiveness of early educational intervention. *Science*, 333, 975–978. 21852490 10.1126/science.1204534

[cit0002] Belsley D. A., Kuh E., & Welsch R. E. (1980). *Regression diagnostics: Identifying influential data and sources of collinearity*. New York, NY: Wiley.

[cit0003] Bloom H. S., Hill C. J., Black A. R., & Lipsey M. W. (2008). Performance trajectories and performance gaps as achievement effect-size benchmarks for educational interventions. *Journal of Research on Educational Effectiveness*, 1, 289–328.

[cit0004] Burchinal M., Vandergrift N., Pianta R., & Mashburn A. (2010). Threshold analysis of association between child care quality and child outcomes for low-income children in pre-kindergarten programs. *Early Childhood Research Quarterly*, 25, 166–176.

[cit0005] Camilli G., Vargas S., Ryan S., & Barnett W. S. (2010). Meta-analysis of the effects of early education interventions on cognitive and social development. *Teachers College Record*, 112, 579–620.

[cit0006] Candelaria C. A., & Shores K. A. (2015, 11). *The sensitivity of causal estimates from court-ordered finance reform on spending and graduation rates* (Center for Education Policy Analysis Working Paper, 16-05). Retrieved from https://cepa.stanford.edu/sites/default/files/shores_candelaria_causal_estimate.pdf

[cit0007] Chetty R., Friedman J. N., & Rockoff J. E. (2014). Measuring the impacts of teachers II: Teacher value-added and student outcomes in adulthood. *American Economic Review*, 104, 2633–2679.

[cit0008] Clotfelter C. T., Ladd H. F., & Vigdor J. L. (2009). Are teacher absences worth worrying about in the United States? *Education Finance and Policy*, 4, 115–149.

[cit0009] Coleman J. S., Campbell E. Q., Hobson C. J., McPartland J., Mood A. M., Weinfeld F. D., & York R. L. (1966). *Equality of educational opportunity*. Washington, DC: Government Printing Office.

[cit0010] Dadey N., & Briggs D. C. (2012). A meta-analysis of growth trends from vertically scaled assessments. *Practical Assessment, Research & Evaluation*, 17(14), 1–13.

[cit0011] Diamond A., Barnett W. S., Thomas J., & Munro S. (2007). Preschool program improves cognitive control. *Science*, 318, 1387. 10.1126/science.1151148 PMC2174918 18048670

[cit0012] Duncan G. J., & Murnane R. J. (Eds.). (2011). *Whither opportunity? Rising inequality, schools, and children’s life chances*. New York, NY: Russell Sage Foundation.

[cit0013] Duncan G. J., & Sojourner A. J. (2013). Can intensive early childhood intervention programs eliminate income-based cognitive and achievement gaps? *Journal of Human Resources*, 48, 945–968. 25620809 10.3368/jhr.48.4.945 PMC4302948

[cit0014] Elder T. E., & Lubotsky D. H. (2009). Kindergarten entrance age and children’s achievement impacts of state policies, family background, and peers. *Journal of Human Resources*, 44, 641–683.

[cit0015] Fahle E. M., Shear B. R., Kalogrides D., Reardon S. F., DiSalvo R., & Ho A. D. (2018). *Stanford Education Data Archive: Technical documentation Version 2.1*. Retrieved from https://stacks.stanford.edu/file/druid:db586ns4974/SEDA_documentation_v21.pdf

[cit0016] Goldhaber D. (2007). Everyone’s doing it, but what does teacher testing tell us about teacher effectiveness? *Journal of Human Resources*, 42, 765–794.

[cit0017] Hanushek E. A. (2003). The failure of input-based schooling policies. *Economic Journal*, 113, 64–98.

[cit0018] Hanushek E. A., & Rivkin S. G. (2010). Generalizations about using value-added measures of teacher quality. *American Economic Review*, 100, 267–271.

[cit0019] Hanushek E. A., Peterson P. E., Talpey L. M., & Woessmann L. (2019, 3). *The unwavering SES achievement gap: Trends in U.S. student performance* (NBER Working Paper No. w25648) Cambridge, MA: National Bureau of Economic Research.

[cit0020] Heckman J., Pinto R., & Savelyev P. (2013). Understanding the mechanisms through which an influential early childhood program boosted adult outcomes. *American Economic Review*, 103, 2052–2086. 24634518 10.1257/aer.103.6.2052 PMC3951747

[cit0021] Hill C. J., Gormley W. T. Jr., & Adelstein S. (2015). Do the short-term effects of a high-quality preschool program persist? *Early Childhood Research Quarterly*, 32, 60–79.

[cit0022] Hoxby C. M. (2001). All school finance equalizations are not created equal. *Quarterly Journal of Economics*, 116, 1189–1231.

[cit0023] Hyman J. (2017). Does money matter in the long run? Effects of school spending on educational attainment. *American Economic Journal: Economic Policy*, 9, 256–280.

[cit0024] Jackson C. K. (2018, 12). Does school spending matter? The new literature on an old question (NBER Working Paper No. 25368). Retrieved from https://www.nber.org/papers/w25368

[cit0025] Jackson C. K., Johnson R. C., & Persico C. (2016). The effects of school spending on educational and economic outcomes: Evidence from school finance reforms. *Quarterly Journal of Economics*, 131, 157–218.

[cit0026] Johnson R. C., & Jackson C. K. (2017, 6). *Reducing inequality through dynamic complementarity: Evidence from head start and public school spending* (NBER Working Paper No. w23489). Washington, MA: National Bureau of Economic Research.

[cit0027] Kane T. J., Rockoff J. E., & Staiger D. O. (2008). What does certification tell us about teacher effectiveness? Evidence from New York City. *Economics of Education Review*, 27, 615–631.

[cit0028] Kornrich S., & Furstenberg F. (2013). Investing in children: Changes in parental spending on children, 1972-2007. *Demography*, 50, 1–23. 22987208 10.1007/s13524-012-0146-4

[cit0029] Kozol J. (1967). *Death at an early age: The destruction of the hearts and minds of Negro children in the Boston Public Schools*. Boston, MA: Houghton Mifflin.

[cit0030] Kozol J. (1991). *Savage inequalities: Children in America’s schools*. New York, NY: Crown.

[cit0031] Lafortune J., Rothstein J., & Schanzenbach D. W. (2018). School finance reform and the distribution of student achievement. *American Economic Journal: Applied Economics*, 10(2), 1–26.

[cit0032] Magnuson K., & Duncan G. J. (2016). Can early childhood interventions decrease inequality of economic opportunity? *Russell Sage Foundation Journal of the Social Sciences: RSF*, 2(2), 123–141. 30135867 10.7758/RSF.2016.2.2.05 PMC6100797

[cit0033] Magnuson K. A., Meyers M. K., & Waldfogel J. (2007). Public funding and enrollment in formal child care in the 1990s. *Social Service Review*, 81, 47–83.

[cit0034] Magnuson K. A., Ruhm C., & Waldfogel J. (2007). Does pre-kindergarten improve school preparation and performance? *Economics of Education Review*, 26, 33–51.

[cit0035] Magnuson K. A., & Waldfogel J. (2005). Early childhood care and education: Effects on ethnic and racial gaps in school readiness. *Future of Children*, 15(2), 169–196. 16130546 10.1353/foc.2005.0005

[cit0036] Magnuson K., & Waldfogel J. (2016). Trends in income-related gaps in enrollment in early childhood education: 1968 to 2013. *AERA Open*, 2(2), 1–13. 30159359 10.1177/2332858416648933 PMC6110399

[cit0037] McCoy D. C., Connors M. C., Morris P. A., Yoshikawa H., & Friedman-Krauss A. H. (2015). Neighborhood economic disadvantage and children’s cognitive and social-emotional development: Exploring Head Start classroom quality as a mediating mechanism. *Early Childhood Research Quarterly*, 32, 150–159. 25937703 10.1016/j.ecresq.2015.04.003 PMC4413938

[cit0038] Miller R. T., Murnane R. J., & Willett J. B. (2008). Do teacher absences impact student achievement? Longitudinal evidence from one urban school district. *Educational Evaluation and Policy Analysis*, 30, 181–200.

[cit0039] Owens A. (2016). Inequality in children’s contexts: Income segregation of households with and without children. *American Sociological Review*, 81, 549–574.

[cit0040] Owens A., Reardon S. F., & Jencks C. (2016). Income segregation between schools and school districts. *American Educational Research Journal*, 53, 1159–1197.

[cit0041] Papay J. P., & Kraft M. A. (2015). Productivity returns to experience in the teacher labor market: Methodological challenges and new evidence on long-term career improvement. *Journal of Public Economics*, 130, 105–119.

[cit0042] Pianta R. C., Barnett W. S., Burchinal M., & Thornburg K. R. (2009). The effects of preschool education: What we know, how public policy is or is not aligned with the evidence base, and what we need to know. *Psychological Science in the Public Interest*, 10, 49–88. 26168354 10.1177/1529100610381908

[cit0043] Piketty T., & Saez E. (2003). Income inequality in the United States, 1913–1998. *Quarterly Journal of Economics*, 118, 1–41. (With tables updated through 2014 at http://eml.berkeley.edu/~saez/TabFig2014prel.xls.)

[cit0044] Raudenbush S. W., Bryk A. S., & Congdon R. (2012). HLM 7 for Windows. Skokie, IL: Scientific Software International.

[cit0045] Reardon S. F. (2011a, 9). Measures of income segregation (Unpublished Working Paper). Retrieved from http://inequality.stanford.edu/sites/default/files/reardon_measures-income-seg.pdf

[cit0046] Reardon S. F. (2011b). The widening academic achievement gap between the rich and the poor: New evidence and possible explanations In Duncan J. & Murnane R. J. (Eds.), *Whither opportunity? Rising inequality, schools, and children’s life chances* (pp. 91–116). New York, NY: Russell Sage Foundation.

[cit0047] Reardon S. F. (2019). Educational opportunity in early and middle childhood: Using full population administrative data to study variation by place and age. *Russell Sage Foundation Journal of the Social Sciences: RSF*, 5(2), 40–68. 31168469 10.7758/RSF.2019.5.2.03 PMC6545991

[cit0048] Reardon S. F., & Bischoff K. (2011). Income inequality and income segregation. *American Journal of Sociology*, 116, 1092–1153. 10.1086/657114 21648248

[cit0049] Reardon S. F., Bischoff K., Owens A., & Townsend J. B. (2018). Has income segregation really increased? Bias and bias correction in sample-based segregation estimates. *Demography*, 55, 2129–2160. 30328018 10.1007/s13524-018-0721-4

[cit0050] Rockoff J. E. (2004). The impact of individual teachers on student achievement: Evidence from panel data. *American Economic Review*, 94, 247–252.

[cit0051] Siegel-Hawley G. (2013). Educational gerrymandering? Race and attendance boundaries in a demographically changing suburb. *Harvard Educational Review*, 83, 580–612.

[cit0052] Sirin S. R. (2005). Socioeconomic status and academic achievement: A meta-analytic review of research. *Review of Educational Research*, 75, 417–453.

[cit0053] Valentino R. (2018). Will public pre-K really close achievement gaps? Gaps in prekindergarten quality between students and across states. *American Educational Research Journal*, 55, 79–116.

[cit0054] Wong V. C., Cook T. D., Barnett W. S., & Jung K. (2008). An effectiveness-based evaluation of five state pre-kindergarten programs. *Journal of Policy Analysis and Management*, 27, 122–154.

